# Evaluation of genome-wide chromatin library of Stat5 binding sites in human breast cancer

**DOI:** 10.1186/1476-4598-4-6

**Published:** 2005-02-01

**Authors:** Matthew J LeBaron, Jianwu Xie, Hallgeir Rui

**Affiliations:** 1 Department of Oncology, Lombardi Comprehensive Cancer Center, Georgetown University, Washington DC, USA

## Abstract

**Background:**

There is considerable interest in identifying target genes and chromatin binding sites for transcription factors in a genome-wide manner. Such information may become useful in diagnosis and treatment of disease, drug target identification, and for prognostication. In cancer diagnosis, patterns of transcription factor binding to specific regulatory chromatin elements are expected to complement and enhance current diagnostic predictions of tumor behavior based on protein and mRNA analyses. Signal transducer and activator of transcription-5 (Stat5) is a cytokine-activated transcription factor implicated in growth and progression of many malignancies, including hematopoietic, prostate, and breast cancer. We have explored immunoaffinity purification of Stat5-bound chromatin from breast cancer cells to identify Stat5 target sites in an unbiased, genome-wide manner.

**Results:**

In this report, we evaluate the efficacy of a Stat5-bound chromatin library to identify valid Stat5 chromatin binding sites within the oncogenome of T-47D human breast cancer cells. A general problem with cloning of immunocaptured, transcription factor-bound chromatin fragments is contamination with non-specific chromatin. However, using an optimized strategy, five out of ten randomly selected clones could be experimentally verified to bind Stat5 both *in vitro *and *in vivo *as tested by electrophoretic mobility shift assay and chromatin immunoprecipitation, respectively. While there was no binding to fragments lacking a Stat5 consensus binding sequence, presence of a Stat5 binding sequence did not assure binding.

**Conclusion:**

A chromatin library coupled with experimental validation may productively identify novel *in vivo *Stat5 chromatin binding sites in cancer, including abnormal regulatory sites in tumor-specific neochromatin.

## Background

Transcription factors function uniquely at the interface of the genome and the proteome. A significant portion of transcription factors serve not only as executors of gene transcription programs, but also as biochemical sensors of extracellular stimuli. For instance, members of the nuclear receptor family are directly activated by lipophilic extracellular ligands, and transcription factors of the Smad and Stat families are activated by phosphorylation in response to cytokine stimulation of cell surface receptors. Chromatin-bound transcription factors that act both as sensors of extracellular cues and as transcriptional effectors carry exceptional instructive value about the biological state of individual cells. Their high biological information value makes such factors particularly attractive for use as markers to predict disease activity and outcome, as well as predictive markers of disease-responsiveness to drugs.

Based on related and broader rationale, the second phase of the human genome project, ENCODE (*ENC*yclopedia *O*f *D*NA *E*lements), has been initiated with the ambitious goal of identifying all regulatory elements of the human genome, including chromatin binding sites for individual transcription factors [1]. In fact, several transcription factors are prognostic biomarkers in cancer, including estrogen and progesterone receptors [2] and signal transducers and activators of transcription (Stats) [3,4]. However, as a result of tumor-specific alterations in chromatin accessibility and structure, individual transcription factors may regulate distinct gene sets in different tumor specimens. Specifically, genes that are actively regulated vary as a result of chromatin structure, DNA methylation, histone modifications, and the presence of additional cofactors. Tumor-specific patterns of transcription factor binding to target chromatin are expected to enhance diagnostic information beyond what is achieved through protein and mRNA analyses. Such added diagnostic information may directly improve disease prognostication and prediction of tumor responsiveness to therapy.

Our laboratory is particularly interested in the role of transcription factor Stat5 in human breast cancer, which is associated with favorable prognosis, especially in early stage malignancy [3]. Stat5 belongs to the Stat transcription factor family, which represents latent cytoplasmic transcription factors that are activated by phosphorylation of a conserved tyrosine residue in response to extracellular cytokines and hormones, such as prolactin, growth hormone, erythropoietin, and several interleukins. Basal activation of Stat5 has been shown in healthy breast epithelial cells [5] and in many early stage breast cancers, but is gradually lost during metastatic progression [3]. Furthermore, active Stat5 correlated with higher histological differentiation and reduced mitotic rate [6]. Additional evidence suggests that Stat5 may actively inhibit metastatic progression by promoting homotypic adhesion and inhibiting tumor cell scattering [7].

Based on technical progress with immunocapture of transcription factor-bound chromatin fragments, genome-wide mapping of interaction sites may be achieved either by hybridization of captured DNA to linear microarrays of genomic DNA, or by cloning and sequencing of captured chromatin fragments. Microarray-based hybridization has been successfully used in the small yeast genome [8], but high cost and technical hurdles remain for human genome-wide DNA arrays at sufficient nucleotide resolution. Early efforts have focused on medium resolution arrays of restricted portions of the genome, such as the small chromosome 22 [9], arrays of classical promoter regions immediately upstream of transcriptional start sites [10], and arrays that contain CpG island clones [11]. In contrast, generation of a genome-wide library of transcription factor-bound chromatin fragments, a chromatin library, represents an inclusive and unbiased approach to the entire human genome [12]. Chromatin libraries also hold the potential to identify transcription factor binding to abnormal, tumor-specific neochromatin arising from genomic instability. However, progress has been hampered by a high degree of non-specific capture of irrelevant chromatin fragments and lack of methods for effective validation of captured sequences.

The purpose of the work described here is to optimize parameters to generate and validate a chromatin library for genome-wide identification of Stat5 target chromatin in human breast cancer. We identify novel Stat5 binding sites from a genome-wide chromatin library and validate the sites by prolactin-inducible Stat5 binding by electrophoretic mobility shift assay (EMSA) and chromatin immunoprecipitation (ChIP).

## Results and Discussion

In contrast to transcription factors that bind to chromatin in a constitutive manner, Stat5 is a latent cytoplasmic transcription factor that is activated by tyrosine phosphorylation and binds tightly to DNA in response to extracellular cytokines, such as prolactin [13]. We used the well-differentiated, estrogen receptor positive T-47D human breast cancer cell line, which maintains robust prolactin-induced Stat5 activation [5,14], to generate a library of Stat5-bound chromatin fragments.

**Figure 1:**
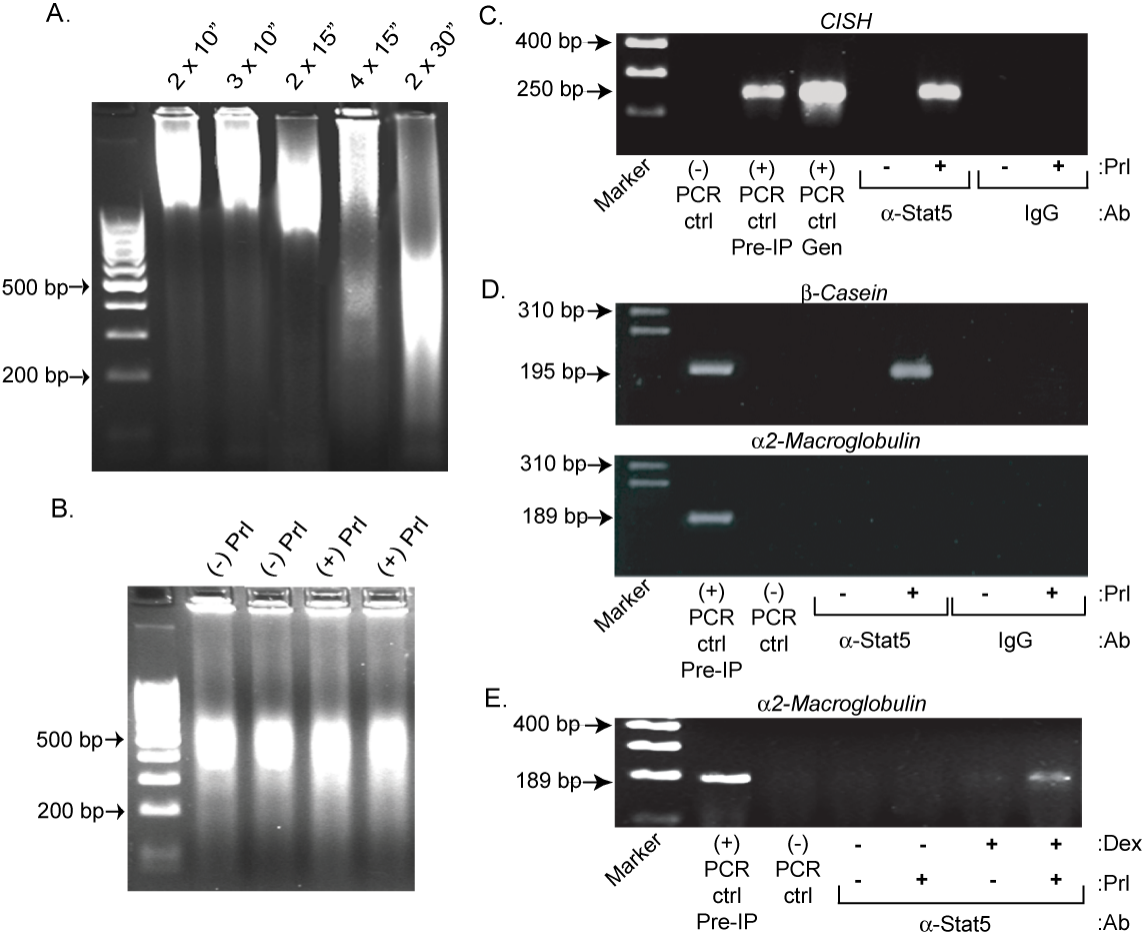
**Specificity of immunocapture of Stat5-bound chromatin in T-47D human breast cancer cells**. *A. Optimization of sonication parameters for shearing genomic DNA in T-47D cells*. Sonication of formaldehyde-fixed cells for two 30 s pulses yielded ~400 bp chromatin fragments. The number and time of sonication pulses are listed above each lane. *B. Prolactin (PRL) pretreatment did not affect chromatin fragmentation*. T-47D cells were treated with (+) or without (-) 10 nM PRL for 30 min, fixed, sonicated (2 × 30 s), and a portion of the pre-IP samples were separated by agarose gel electrophoresis. *C-E. Validation of quality of immunocaptured, Stat5-bound chromatin pool by ChIP analysis of known Stat5 target genes*. Cells were incubated with (+) or without (-) 10 nM PRL for 30 min, and processed as described in *Methods*. Non-specific IgG was used as a negative IP control and PCR was performed using primers designed to specifically amplify the known Stat5 response element. PCR products shown are representative of at least two separate amplifications of at least two independently generated pools of Stat5-chromatin complexes. (-) PCR ctrl indicates a negative (no template) PCR control, (+) PCR ctrl Pre-IP indicates a positive PCR control performed on the pre-immunoprecipitated (input) DNA, and (+) PCR ctrl Gen indicates a positive PCR control performed on purified T-47D genomic DNA. PRL activated Stat5 specifically associated with the promoter of *CISH *(C) and *β-Casein *(D – upper panel), but not *α2-Macroglobulin *(D – lower panel), unless cells had been pretreated for four days with glucocorticoid (E – 1 μM dexamethasone; Dex).

Sonication is necessary to shear genomic DNA into fragments that can be easily manipulated for PCR amplification, cloning, and sequencing. Fragments of approximately 400 base pairs (bp) allow for a complete sequencing read-through and are of sufficient size to localize the fragment within the human genome with a high degree of statistical certainty. Optimal shearing of chromatin from formaldehyde-fixed T-47D cells into approximately 400 bp fragments was established empirically (Figure 1A, lane 5). This target size of chromatin fragments was confirmed by agarose gel electrophoresis of two parallel sets of sonicates of cells treated with or without prolactin for 30 min (Figure 1B), prior to immunocapture of Stat5-bound fragments. An antibody that recognizes the highly homologous Stat5a and Stat5b isoforms [13] was used to capture Stat5-bound chromatin as detailed in *Methods*. Before subcloning of the captured chromatin fragments, the specificity of immunocapture was verified by analysis of binding to known human Stat5 target chromatin. In particular, we took advantage of earlier work that has identified a group of Stat5 regulated genes that have been shown to contain the Stat5 consensus sequence, TTCNNNGAA, in the traditional promoter element [13,15]. Aliquots of the captured chromatin pool were amplified by PCR using oligonucleotide primers flanking Stat5 binding sites within the gene promoters of *Cytokine-Inducible SH2 Protein *(*CISH*), *β-Casein*, and *α2-Macroglobulin*. Due to the average chromatin fragment size of 400 bp, primers were designed to yield shorter PCR products of 200 – 300 bp.

Stat5 was inducibly associated with the promoter of the *CISH *gene response element in T-47D cells (Figure 1C). The capture was specific, since binding was only detected in prolactin-stimulated cells, and only when Stat5 antibody and not control IgG was used. PCR amplification of intact genomic DNA is shown as a control to verify the specificity of the PCR reaction, in addition to amplification of the pre-immunoprecipitation chromatin fragment pool (input DNA). Likewise, Stat5 was inducibly and specifically bound to the *β-Casein *gene promoter in T-47D cells (Figure 1D, upper panel). In contrast, Stat5 did not associate with the Stat5-response element of the *α2-Macroglobulin *gene (Figure 1D, lower panel), a gene reportedly responsive to Stat5 in liver [16], uterine stromal cells [17], and ovarian cells [18,19]. However, pretreatment of T-47D cells with the glucocorticoid hormone analog, dexamethasone, for four days prior to Stat5 activation made the *α2-Macroglobulin *promoter accessible to Stat5 binding (Figure 1E). It has been well established that glucocorticoids play a vital role in many cell types and cell processes, including mammary differentiation. In fact, several genes have been shown to be regulated by cooperative Stat5-glucocorticoid receptor interactions [20-23]. In summary, based on Stat5 inducibility and antibody specificity in testing of known Stat5 chromatin interaction sites, we concluded that the conditions for effective immunocapture of Stat5-bound chromatin from T-47D cells were established.

**Figure 2:**
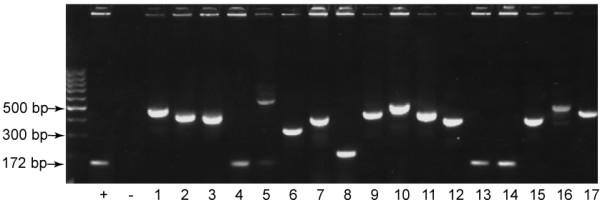
**Cloned Stat5-bound chromatin fragments from PRL-stimulated T-47D breast cancer cells**. Immunoprecipitated chromatin fragments were modified for cloning into a TA cloning vector and were transformed into electrocompetent bacteria. PCR was performed directly on white bacterial colonies using primers flanking the cloning site and was cycled 36 times. Samples were run on an agarose gel and analyzed for the presence of insert. (+) denotes vector control (no insert yields a vector-derived 172 bp product); (-) denotes negative PCR control.

The enriched, Stat5-bound chromatin pool was then cloned into a bacterial vector to generate a chromatin library. Because sonication generates random overhangs in double stranded DNA [24], T4 DNA polymerase was first used to blunt-end DNA fragments. Subsequently, a single 3' adenosine residue was added using *Taq *polymerase, and the resulting fragments were ligated into the pCR2.1 TA cloning vector. Transformed bacteria were plated on ampicillin- and S-gal-containing selection plates for blue/white screening. PCR amplification of inserts was performed directly on white bacterial colonies with common primers flanking the vector cloning site. A PCR reaction under standard conditions was used to lyse the bacteria and inactivate endogenous nucleases, cycled 36 times, and the products were separated by agarose gel electrophoresis. Initial analysis of 389 white colonies yielded 185 (48%) insert-containing PCR products. Figure 2 shows a display of PCR products from a run of 17 clones, in which three clones did not contain an insert and 14 clones had inserts of a median size of approximately 300 bp. PCR products containing an insert were purified and sequenced directly without plasmid minipreps in a cost-effective and time-saving manner. BLAST analysis was used to localize sequences to the human genome. Of 185 inserts, 31 (17%) sequences could be unambiguously matched to a location within the human genome. Sequences that could not be localized were either repetitive or did not produce a statistically significant homology to the published human genome.

**Figure 3:**
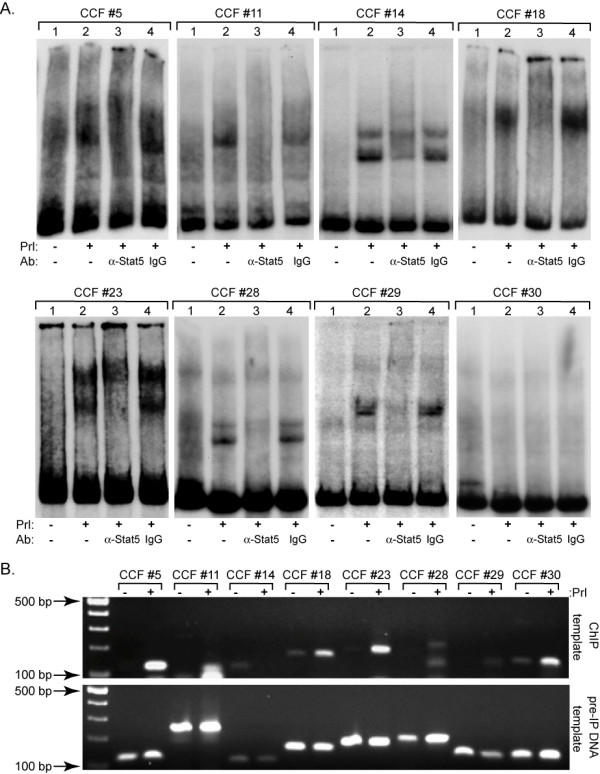
**Validation of cloned Stat5-bound chromatin fragments from PRL-stimulated T-47D breast cancer cells**. *A. PRL-activated Stat5 binds *in vitro *to 7 of 10 (8 shown) Cloned Chromatin Fragments (CCF) obtained from a Stat5-chromatin library as shown by EMSA*. Nuclear extracts from confluent T-47D cells overexpressing Stat5 and incubated with (+) or without (-) 10 nM human PRL for 30 min were used. Prolactin-induced protein-DNA complexes are indicated by the retarded migration of probe in the (+) PRL lanes for each of the CCFs. Supershift reactions, or band depletions, are indicated by the antibody (Ab) added and signify a specific Stat5-DNA interaction when compared to the supershift control, non-specific, purified IgG. *B. Validation of *in vivo *Stat5 binding to immunocaptured Cloned Chromatin Fragments (CCFs) by ChIP assay*. T-47D cells were incubated with (+) or without (-) PRL as described and Stat5-DNA complexes were enriched by chromatin immunoprecipitation. PCR primers specific for each CCF were designed and used for amplification of two independently generated pools of Stat5-chromatin interaction sites and PCR was performed twice on each pool; a representative experiment is shown. CCFs #5, #18, #23, #28, #29, and #30 were consistently positive for *in vivo *Stat5-DNA binding (upper panel). Positive PCR controls were performed on pre-IP template (lower panel).

To validate the quality of the chromatin library, ten clones were randomly selected and first tested for ability to bind activated Stat5 from nuclear extracts of T-47D cells *in vitro *by EMSA. Stat5 binds to the consensus sequence TT(N5)AA and to relatively conservative variations, with TTC(N3)GAA considered to be optimal [13,15]. Typically, Stat5-DNA interaction by EMSA is performed on synthetic oligonucleotides of 20–40 bp size [25]. To effectively determine whether Stat5 interacts directly with large immunocaptured chromatin fragments, we established conditions for rapid validation by EMSA on chromatin fragments up to 400 bp in length by reducing gel polyacrylamide concentration to 3% and using amplification and isotope labeling by PCR directly from the bacterial clones. Stat5 binding *in vitro *was detected in seven of the ten chromatin fragments, as evidenced by prolactin-inducible DNA-binding complexes that could be supershifted by a specific anti-Stat5 antibody, but not by non-specific IgG (Figure 3A). Negative data are only presented for one cloned chromatin fragment (CCF #30) of the three non-binding fragments (CCF # 21, # 25, and #30).

As a second and independent means to validate the quality of the Stat5 chromatin library, the same randomly selected fragments were analyzed for inducible Stat5 binding *in vivo *using the ChIP assay. Independent pools of immunocaptured chromatin fragments from T-47D cells were analyzed in which Stat5 was either inactivated by serum deprivation or activated by prolactin. Densitometry of the PCR products was used to verify at least a 2-fold increase in intensity between the (-) prolactin and (+) prolactin samples. In all cases there was no detectable product from the replicate samples that had been immunoprecipitated with a non-specific IgG antibody (data not shown), indicating a specific Stat5-mediated capture of genomic elements. All PCR amplifications were performed at least twice on at least two separate pools of immunoprecipitated genomic elements.

**Figure 4:**
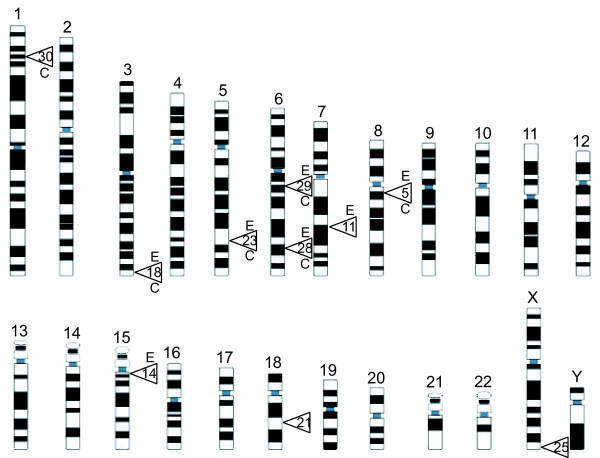
**Genomic localization of cloned Stat5-chromatin interaction sites from T-47D human breast cancer cells**. A karyogram of the human genome illustrates the loci of the cloned Stat5 binding sites. Each site is designated by the CCF number and is localized to its specific area of the genome, based on BLAST alignment. CCFs positive by EMSA are labeled "E", and CCFs positive by ChIP are labeled "C". The genomic localization and two nearest genes of each verified CCF is given by the genomic position of the centrally located nucleotide: *CCF#5*: 8q12.1:58980112 (LOC389663-similar to hypothetical protein; MGC39325-hypothetical protein); *CCF#18*: 3q29:193835263 (FGF12-fibroblast growth factor 12; LOC151963-similar to BcDNA:GH11415 gene product); *CCF#23*: 5q33.1:147357743 (LOC391839-similar to elongation factor 1 gamma; SPINK5-serine protease inhibitor, Kazal type, 5); *CCF#28*: 6q24.1:142177080 (LOC340149-hypothetical protein; NMBR-neuromedin B receptor); *CCF#29*: 6q13:74774694 (CD109 antigen); SLC17A5-solute carrier family 17 (anion/sugar transporter) member 5). Other CCFs include: *CCF#11*: 7q31.1:111547940; *CCF#14*: 15q11.2:21265800; *CCF#21*: 18q21.2:49639649; *CCF#25*: Xq28:153691722; *CCF#30*: 1p34.1:43842775.

Of the seven chromatin fragments that were positive for *in vitro *Stat5 binding by EMSA, five (CCFs #5, #18, #23, #28, and #29) were also consistently positive for *in vivo *Stat5 binding by ChIP assay (Figure 3B, upper panel). In addition, CCF #30 was positive by ChIP, but not by EMSA, possibly reflecting indirect binding via other proteins. Conversely, CCFs #11 and #14, which bound Stat5 *in vitro *by EMSA, were both negative by ChIP. Corresponding PCR products from the pre-IP DNA is shown (Figure 3B, lower panel) and no product was detected in samples immunoprecipitated with non-specific IgG (data not shown). Due to sequence complexity, fragment-specific flanking primers could not be designed for fragments CCF #21 and #25, neither of which bound Stat5 in EMSA. The localization data and binding validation of the ten clones are summarized in Figure 4. Each of the CCFs that bound Stat5 by EMSA contained at least one broad consensus TT(N5)AA site as expected. Correspondingly, of the three EMSA-negative CCFs, #21 and #25 lacked consensus binding sites, while a single TT(N5)AA site was present in CCF #30. Furthermore, the five CCFs verified to bind both by EMSA and ChIP may be involved in transcriptional control of nearby genes (Figure 4), or alternatively, control transcription of small regulatory RNAs [26].

## Conclusions

While the present work was being completed, an independent report also identified Stat5 binding sites using a genome-wide approach in mouse lymphoma cells [27], although direct binding by EMSA was not verified. We conclude that unbiased, genome-wide strategies can now be used to identify novel Stat5 binding sites by cloning immunocaptured chromatin fragments. At the current efficacy, approximately 20% of cloned Stat5-immunocaptured fragments from T-47D breast cancer cells could be localized within the normal human genome, and Stat5 binding *in vitro *and *in vivo *was confirmed in approximately half of those. Further reductions in capture of non-specific chromatin, combined with refinements in cloning procedures [28], reduced sequencing cost, direct and high-throughput sequencing from bacterial colonies, and direct labeling of PCR products for EMSA testing, will allow streamlining of the procedure for genome-wide identification of Stat5-chromatin interaction sites. Ongoing efforts are also exploring whether some of the clones with only weak homology to the normal human genome represent Stat5-bound neochromatin unique to the cancer cells as a result of genomic instability.

## Methods

### Cell Culture and Hormones

The human breast cancer cell line T-47D was grown to confluence in RPMI-1640 medium (Biofluids, Rockville, MD) supplemented with 10% fetal bovine serum, 2 mM L-glutamine, and penicillin-streptomycin (50 IU/ml and 50 μg/ml, respectively) at 37°C with 5% CO_2_. Human prolactin was kindly provided by Dr. A.F. Parlow under the sponsorship of the NIDDKs National Hormone & Peptide Program. Dexamethasone (Dex) was purchased from Sigma Chemical Co. (St. Louis, MO).

### Chromatin Immunoprecipitation

After grown to confluence (~2 × 10^7 ^cells/T175 cm^2 ^flask), T-47D cells were serum starved for 24 h and then treated with or without 10 nM hPRL for 30 min. For prodifferentiation experiments with glucocorticoid pretreatment, confluent cultures were maintained in serum-free RPMI-1640 with 1 μM Dex dissolved in DMSO or in DMSO alone for 96 hours, then stimulated with or without PRL for 30 min. Proteins were then crosslinked to the chromatin by the addition of formaldehyde (Fisher Scientific, Fairlawn, NJ) to a final concentration of 1% and incubated for 30 min at 37°C. The cells were rinsed, scraped, and pelleted in ice-cold PBS with 1 mM PMSF, 2 μg/ml aprotinin, and 2 μg/ml pepstatin A. Cell pellets were resuspended in 400 μl lysis buffer (1% SDS, 10 mM EDTA, 50 mM Tris-HCl, pH 8.0, 1 mM PMSF, 2 μg/ml aprotinin, and 2 μg/ml pepstatin A) and the lysates were sonicated using a Sonic Dismembrator (Fisher Scientific, Pittsburgh, PA), fitted with a tapered microtip set to an amplitude of 50% and were pulsed twice for 30 s. Debris was pelleted by centrifugation for 10 min at 13,200 RPM at 4°C. The supernatants were then diluted 10-fold in IP buffer (0.1% SDS, 1.1% Triton-X, 1.2 mM EDTA, 16.7 mM Tris-HCl, pH 8.1, 16.7 mM NaCl, 1 mM PMSF, 2 μg/ml aprotinin, and 2 μg/ml pepstatin A) and 1% was set aside for Pre-IP or input sample. The solution was then pre-cleared with beads (50% protein A-sepharose, 1 mg/ml poly dI-dC, 0.1% BSA, in TE, pH 7.4) for 30 min at 4°C. Next, the samples were incubated overnight with Stat5 antibody (N-20, Santa Cruz Biotechnology) or IgG IP-control followed by immunoprecipitation with pre-incubated beads. The samples (beads) were then washed once with each of these buffers in the following order: Wash Buffer 1 (0.1% SDS, 1% Triton-X, 2 mM EDTA, 20 mM Tris-HCl, pH 8.0, 150 mM NaCl), Wash Buffer 2 (0.1% SDS, 1% Triton-X, 2 mM EDTA, 20 mM Tris-HCl, pH 8.0, 500 mM NaCl), Wash Buffer 3 (0.25 M LiCl, 1% NP-40, 1% Na-deoxycholate, 10 mM Tris-HCl, pH 8.0, 1 mM EDTA), then twice with Wash Buffer 4 (TE, pH 8.0). The samples were eluted from the beads in 1% SDS and 0.1 M NaHCO_3_. Next, the cross-links were reversed with 0.2 M NaCl overnight at 65°C followed by protein digestion with proteinase K for 1 hour at 45°C. The DNA was recovered by phenol:chloroform extraction and ethanol precipitation. This pool was then used as PCR template or could be blunted with T4 DNA polymerase (New England Biolabs, Beverly, MA) at 37°C for 5 minutes under standard conditions for uniform cloning. *Taq *DNA polymerase (New England Biolabs) was then used to add a 3' A overhang for cloning into a TOPO TA cloning kit (Invitrogen, Carlsbad, CA). The ligated vectors were transformed into electrocompetent bacteria, DH-5*α*E (Invitrogen), using the manufacturer's recommendations for transformation in a Bio-Rad Gene-Pulser (Hercules, CA), and plated on S-Gal/Ampicillin plates (Sigma Chemical Co., St. Louis, MO). Positive clones by blue-white screening were then analyzed directly by colony PCR in a standard reaction with vector-specific M13 and T7 primers that flank the cloning site and were incubated at 94°C for 4 min and cycled at 94°C for 30 s, 55°C for 45 s, and 72°C for 30 s. Colonies with quantifiable inserts were then purified (QiaQuick PCR purification kit, Qiagen, Valencia, CA), sequenced, and localized using BLAST (NCBI, NIH, Bethesda, MD).

### Validation using known Stat5 responsive genes

The following primers were designed to flank the Stat5 binding site in the promoter of the following genes: *α2-Macroglobulin *forward 5' TTT AGC CCT CCA GGG ATT CT 3', reverse 5' CAA TCC ATC TGG TCC CAA AC 3'; *β-Casein *forward 5' GGA GAA ACA GTT TGC CTC ACA 3', reverse 5' CCT AGT GGG GCC TTG AGA TT 3'; *CISH *forward 5' CTA TTG GCC CTC CCC GAC 3', reverse 5' AGC TGC TGC CTA ATC CTT TG 3'. PCR amplifications shown are representative of at least 2 separate amplifications of at least 2 independently generated immunocaptured pools of Stat5-chromatin complexes.

### Preparation of cellular extracts for EMSA

After reaching confluence, T-47D cells were infected with adenovirus containing wild type Stat5 at a Multiciplity Of Infection (MOI) = 6.67, as described previously [29]. Parallel samples of T-47D cells were not exposed to adenovirus as a mock infection (standard control). After infection, the cells were cultured in serum-free medium for 24 hours, then stimulated for 30 min with 10 nM hPRL, the culture medium was removed, and the cells were collected as described above. Nuclear lysates were collected as described previously [5,25].

### Generation of radiolabeled DNA probes

Radiolabeled products were generated by PCR in a 10 μl reaction under standard conditions with the addition of 0.25 μl *α*^32^P dATP (10 mCi/ml; Amersham-Pharmacia, Piscataway, NJ). Initially the samples were incubated at 94°C for 1 min, then cycled 36 times at 94°C for 30 s, 55°C for 30 s, and 72°C for 30 s. The reaction was then held at 72°C for 5 min following the cycling to allow for product fill-in and addition of a 3' terminal "A". After completion of the cycling, the PCR products were purified using the Qiagen PCR purification kit, according to manufacturer's instructions. The final products were eluted in 50 μl and stored at -20°C until use.

### DNA-protein binding reaction

The DNA-protein binding reactions were performed in a 10 μl mixture containing 3 μl of nuclear extract from the respective sample, and 1 μg of double-stranded poly dI:dC (Boehringer Mannheim, Indianapolis, IN), as previously described [25]. After 1 h on ice, samples (with 1 ng specific anti-Stat5 antibody (Santa Cruz Biotechnology), or 1 ng non-specific, purified IgG (Sigma), or no antibody) were incubated with 2.0 μl ^32^P-labeled PCR probe and incubated for 20 min at room temperature. The samples were then resolved in a 3% non-denaturing polyacrylamide gel, as previously described [25].

### ChIP in vivo validation

As an independent validation technique, we designed primers specific to each cloned chromatin fragment (CCF) then performed PCR on a separately-generated enriched pool of immunoprecipitated Stat5-chromatin interaction sites. The following primers were used for PCR: *CCF #5 *forward 5' TGA CAT CAG TGA GAG TGG AGG 3', reverse 5' TGA GGC TGT AAT GTC ACT CAG AA 3'; *CCF #11 *forward 5' GGA CAC ATC CAC TAC TGC CA, reverse 5' AAA AGA AGT GCA GTT CAG GAT AA 3'; *CCF #14 *forward 5' GCC TGT GTG ATA TCA TAT GGA AAG 3', reverse 5' TTT CCA AGA ACT TAT CAG AAT GAC TT 3'; *CCF #18 *forward 5' TCA GTC TTC CTC TTT CCC CA 3', reverse 5' CGG CTT ACA TTC TGT GTC GC 3'; *CCF #23 *forward 5' CAT ATC TGT CTA CAA ATA ACA GTT CCC 3', reverse 5' AAT AGA GGG CAG AGT TAA GAA TCA AA 3'; *CCF #28 *forward 5' AAG CCT GAA AAG AAA AAT CTC A 3', reverse 5' CCT GAC ACA TCC AGA CTT GG 3'; *CCF #29 *forward 5' CAG ACG TTT GCT GGA AGA TAT G 3', reverse 5' TTG TTC CAT GAA ACC AGG G 3'; *CCF #30 *forward 5' AAG GCC ATT GAA GTG AGG TG 3', reverse 5' CCA TCA GCC AGT CAT TGA AG 3'. The PCR was performed with standard conditions using template immunoprecipitated from cells treated with or without prolactin to indicate Stat5 inducibility and was executed at least twice on each of 2 separate pools.

## Authors' contributions

MJL carried out the experimental studies, participated in the design of the study, and drafted the manuscript. JX participated in the design of the study and scientific discussion of the results. HR conceived and participated in the design of the study, contributed in the evaluation of the results, and in the preparation of the manuscript. All authors read and approved the final manuscript.

## References

[B1] (2004). The ENCODE (ENCyclopedia Of DNA Elements) Project. Science.

[B2] Hayes DF, Isaacs C, Stearns V (2001). Prognostic factors in breast cancer: current and new predictors of metastasis. J Mammary Gland Biol Neoplasia.

[B3] Nevalainen MT, Xie J, Torhorst J, Bubendorf L, Haas P, Kononen J, Sauter G, Rui H (2004). Signal transducer and activator of transcription-5 activation and breast cancer prognosis. J Clin Oncol.

[B4] Widschwendter A, Tonko-Geymayer S, Welte T, Daxenbichler G, Marth C, Doppler W (2002). Prognostic significance of signal transducer and activator of transcription 1 activation in breast cancer. Clin Cancer Res.

[B5] Nevalainen MT, Xie J, Bubendorf L, Wagner KU, Rui H (2002). Basal activation of transcription factor signal transducer and activator of transcription (Stat5) in nonpregnant mouse and human breast epithelium. Mol Endocrinol.

[B6] Cotarla I, Ren S, Zhang Y, Gehan E, Singh B, Furth PA (2004). Stat5a is tyrosine phosphorylated and nuclear localized in a high proportion of human breast cancers. Int J Cancer.

[B7] Sultan AS, Xie J, Lebaron MJ, Ealley EL, Nevalainen MT, Rui H (2005). Stat5 promotes homotypic adhesion and inhibits invasive characteristics of human breast cancer cells. Oncogene.

[B8] Ren B, Robert F, Wyrick JJ, Aparicio O, Jennings EG, Simon I, Zeitlinger J, Schreiber J, Hannett N, Kanin E, Volkert TL, Wilson CJ, Bell SP, Young RA (2000). Genome-wide location and function of DNA binding proteins. Science.

[B9] Buckley PG, Mantripragada KK, Benetkiewicz M, Tapia-Paez I, Diaz De Stahl T, Rosenquist M, Ali H, Jarbo C, De Bustos C, Hirvela C, Sinder Wilen B, Fransson I, Thyr C, Johnsson BI, Bruder CE, Menzel U, Hergersberg M, Mandahl N, Blennow E, Wedell A, Beare DM, Collins JE, Dunham I, Albertson D, Pinkel D, Bastian BC, Faruqi AF, Lasken RS, Ichimura K, Collins VP, Dumanski JP (2002). A full-coverage, high-resolution human chromosome 22 genomic microarray for clinical and research applications. Hum Mol Genet.

[B10] Li Z, Van Calcar S, Qu C, Cavenee WK, Zhang MQ, Ren B (2003). A global transcriptional regulatory role for c-Myc in Burkitt's lymphoma cells. Proc Natl Acad Sci U S A.

[B11] Weinmann AS, Yan PS, Oberley MJ, Huang TH, Farnham PJ (2002). Isolating human transcription factor targets by coupling chromatin immunoprecipitation and CpG island microarray analysis. Genes Dev.

[B12] Weinmann AS, Bartley SM, Zhang T, Zhang MQ, Farnham PJ (2001). Use of chromatin immunoprecipitation to clone novel E2F target promoters. Mol Cell Biol.

[B13] Grimley PM, Dong F, Rui H (1999). Stat5a and Stat5b: fraternal twins of signal transduction and transcriptional activation. Cytokine Growth Factor Rev.

[B14] Schaber JD, Fang H, Xu J, Grimley PM, Rui H (1998). Prolactin activates Stat1 but does not antagonize Stat1 activation and growth inhibition by type I interferons in human breast cancer cells. Cancer Res.

[B15] Ehret GB, Reichenbach P, Schindler U, Horvath CM, Fritz S, Nabholz M, Bucher P (2001). DNA binding specificity of different STAT proteins. Comparison of in vitro specificity with natural target sites. J Biol Chem.

[B16] Ripperger JA, Fritz S, Richter K, Hocke GM, Lottspeich F, Fey GH (1995). Transcription factors Stat3 and Stat5b are present in rat liver nuclei late in an acute phase response and bind interleukin-6 response elements. J Biol Chem.

[B17] Prigent-Tessier A, Barkai U, Tessier C, Cohen H, Gibori G (2001). Characterization of a rat uterine cell line, U(III) cells: prolactin (PRL) expression and endogenous regulation of PRL-dependent genes; estrogen receptor beta, alpha(2)-macroglobulin, and decidual PRL involving the Jak2 and Stat5 pathway. Endocrinology.

[B18] Russell DL, Norman RL, Dajee M, Liu X, Henninghausen L, Richards JS (1996). Prolactin-induced activation and binding of stat proteins to the IL-6RE of the alpha 2-macroglobulin (alpha 2M) promoter: relation to the expression of alpha 2M in the rat ovary. Biol Reprod.

[B19] Dajee M, Kazansky AV, Raught B, Hocke GM, Fey GH, Richards JS (1996). Prolactin induction of the alpha 2-Macroglobulin gene in rat ovarian granulosa cells: stat 5 activation and binding to the interleukin-6 response element. Mol Endocrinol.

[B20] Doppler W, Groner B, Ball RK (1989). Prolactin and glucocorticoid hormones synergistically induce expression of transfected rat beta-casein gene promoter constructs in a mammary epithelial cell line. Proc Natl Acad Sci U S A.

[B21] Lechner J, Welte T, Tomasi JK, Bruno P, Cairns C, Gustafsson J, Doppler W (1997). Promoter-dependent synergy between glucocorticoid receptor and Stat5 in the activation of beta-casein gene transcription. J Biol Chem.

[B22] Stoecklin E, Wissler M, Moriggl R, Groner B (1997). Specific DNA binding of Stat5, but not of glucocorticoid receptor, is required for their functional cooperation in the regulation of gene transcription. Mol Cell Biol.

[B23] Wyszomierski SL, Yeh J, Rosen JM (1999). Glucocorticoid receptor/signal transducer and activator of transcription 5 (STAT5) interactions enhance STAT5 activation by prolonging STAT5 DNA binding and tyrosine phosphorylation. Mol Endocrinol.

[B24] Elsner HI, Lindblad EB (1989). Ultrasonic degradation of DNA. DNA.

[B25] Kirken RA, Malabarba MG, Xu J, Liu X, Farrar WL, Hennighausen L, Larner AC, Grimley PM, Rui H (1997). Prolactin stimulates serine/tyrosine phosphorylation and formation of heterocomplexes of multiple Stat5 isoforms in Nb2 lymphocytes. J Biol Chem.

[B26] Cawley S, Bekiranov S, Ng HH, Kapranov P, Sekinger EA, Kampa D, Piccolboni A, Sementchenko V, Cheng J, Williams AJ, Wheeler R, Wong B, Drenkow J, Yamanaka M, Patel S, Brubaker S, Tammana H, Helt G, Struhl K, Gingeras TR (2004). Unbiased mapping of transcription factor binding sites along human chromosomes 21 and 22 points to widespread regulation of noncoding RNAs. Cell.

[B27] Nelson EA, Walker SR, Alvarez JV, Frank DA (2004). Isolation of unique STAT5 targets by chromatin immunoprecipitation-based gene identification. J Biol Chem.

[B28] Roh TY, Ngau WC, Cui K, Landsman D, Zhao K (2004). High-resolution genome-wide mapping of histone modifications. Nat Biotechnol.

[B29] Ahonen TJ, Xie J, LeBaron MJ, Zhu J, Nurmi M, Alanen KA, Rui H, Nevalainen MT (2003). Inhibition of transcription factor Stat5 induces cell death of human prostate cancer cells. J Biol Chem.

